# Angular momentum–scattering angle quantum correlation: a generalized deflection function

**DOI:** 10.1039/c7sc05489k

**Published:** 2018-04-26

**Authors:** P. G. Jambrina, M. Menéndez, F. J. Aoiz

**Affiliations:** a Departamento de Química Física Aplicada , Universidad Autonoma de Madrid , 28049 , Madrid , Spain . Email: pablo.gjambrina@uam.es; b Departamento de Química Física I , Facultad de Ciencias Químicas , Universidad Complutense de Madrid , 28040 Madrid , Spain . Email: aoiz@quim.ucm.es

## Abstract

A natural generalization of the classical deflection function, the functional dependence of the deflection angle on the angular momentum (or the impact parameter), is the joint probability density function of these two quantities, revealing the correlation between them. It provides, at a glance, detailed information about the reaction mechanisms and how changes in the impact parameter affect the product angular distribution. It is also useful to predict the presence of quantum phenomena such as interference. However, the classical angular momentum–scattering angle correlation function has a limited use whenever quantum effects become important. Rigorously speaking, there is not a quantum equivalent of the classical joint distribution, as the differential cross section depends on the coherences between the different values of *J* caused by the cross terms in the expansion of partial waves. In this article, we present a simple method to calculate a quantum analog of this correlation, a generalized deflection function that can shed light onto the reaction mechanism using just quantum mechanical results. Our results show that there is a very good agreement between the quantum and classical correlation functions as long as quantum effects are not all relevant. When this is not the case, it will also be shown that the quantum correlation function is most useful to observe the extent of quantum effects such as interference among different reaction mechanisms.

## Abstract 2

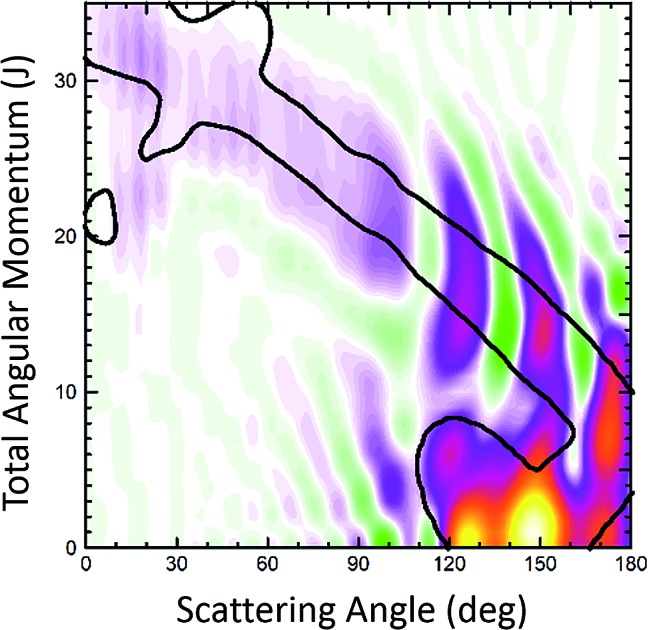
A quantum generalized deflection function is aimed at unravelling quantum effects in chemical reactions.

## 1 Introduction

The main goal of reaction dynamics is to obtain various microscopical properties such as excitation functions or rotational distributions and from them, macroscopical properties such as thermal rate coefficients. Overall, the process is equivalent to determining how microscopical properties govern the macroscopic outcome. Accordingly, it is not enough to reproduce and to predict experimental measurements, but it is also important to unveil detailed reaction mechanisms.

The impact parameter *b* (or the orbital angular momentum *𝓁*) and scattering angle *θ* are the two main variables that are studied to discern reaction mechanisms. The former is related to the asymptote of reactants and is one of the key factors in determining the outcome of a collision,^1^,^2^ as it determines the regions of the potential energy surface (PES) that will be explored during the collision (head-on *vs.* glancing collisions). The scattering angle, in turn, is defined at the product asymptote and provides information about nuclei scrambling during the collision; besides, it is amenable to experimental measurement using cross molecular beams with mass spectrometric universal detection or, more recently, velocity-mapped ion imaging^3^–^6^ or single beam coexpansion such as photoloc^7^ among other techniques. Moreover, from the theoretical point of view, it is relatively straightforward to extract the reaction probability as a function of *J* (opacity function or *P*_r_(*J*)), and the differential cross section (DCS or *σ*_r_(*θ*)) as a function of the scattering angle *θ*. Hence, it is not surprising that *P*_r_(*J*) and DCS are two of the most important quantities used to determine the collision mechanism. However, the knowledge of these two distributions may not be sufficient to characterize the mechanisms. To this purpose, it would be necessary to relate how the initial and final conditions are correlated; specifically, which impact parameters give rise to scattering at certain angles.

To relate the angular momentum and scattering angle, the deflection function (DF), that is, the functional of the deflection angle (*Θ* whose absolute value is *θ*) in terms of the angular momentum, in its classical, semiclassical and quantal versions, has been widely used to explain elastic and inelastic scattering, in particular to understand those features related to glory and rainbow scattering.^1^,^8^,^9^ More recently, Connor and coworkers have devised a quantum deflection function (QDF) in the context of the glory analysis of forward scattering that can also be applied to reactive scattering.^10^–^12^ The QDF, defined as the derivative of the argument of the scattering matrix element with respect to *J*, has proved to be a valuable tool to predict the presence of rainbows. Besides, it could be used to predict interference between nearside and farside scattering. However, the QDF does not consider that a single *J* can correlate with different *θ* which limits its use to predict the presence of different mechanisms.

Within a classical mechanics framework, there is no limitation in the amount of information that may relate the initial and final conditions in a collision. Thus, it is perfectly feasible to go beyond the DF and to determine the joint dependence of the reaction probability as a function of the scattering angle and the impact parameter, the *J*–*θ* correlation function. This sort of generalization of the DF not only contains all the information provided by the *P*_r_(*J*) and the DCS but also, primarily, provides how *J* and *θ* correlate throughout the collision. For reactive scattering, a strong correlation between *J* and *θ* is expected for reactions following a direct mechanism, whereas no or very weak correlation between these variables can be anticipated if the reaction takes place through a long-lived collision complex. Furthermore, discontinuities and different trends in the *J*–*θ* correlation can be associated with different reaction mechanisms, and permit its characterization even for apparently simple reactions.^13^,^14^

The classical joint probability distribution has also been used to predict interference causing oscillations in the DCS.^15^,^16^ Given the wave nature of quantum mechanics (QM), it is expected that when one particle may follow two different pathways with the same outcome, they will interfere. In Young's double-slit experiment,^17^ interference arises when electrons going through two different slits hit the detector. In reaction dynamics we do not need slits and the system itself acts as an interferometer whenever two different *J* could scatter at the same angles and final quantum state.^15^,^16^,^18^ This analogy also explains why a correlation function between *θ* and *J* cannot be calculated using pure quantum mechanical grounds, as it is done in classical calculations. In QM, the angular distribution depends on the coherences between different partial waves, and therefore something apparently as simple as obtaining a rigorous joint probability distribution as a function of *J* and *θ* cannot be computed. This would be similar to disentangling which part of the signal comes from electrons going through one or the other slit in Young's double-slit experiment.

Throughout this article, we will try to circumvent this limitation and propose a new quantum analog to the classical correlation function, *Q*_r_(*θ*,*J*), that will appear as a generalized deflection function (GDF) for the interpretation of quantum scattering results. This new function is a joint quasi-probability distribution of *J* and *θ* that includes all coherences between different partial waves, and whose summation over all partial waves recovers the exact differential cross section. As will be shown *Q*_r_(*θ*,*J*) emerges as a valuable tool to assist in the elucidation of reaction mechanisms, especially when quantum phenomena are important, or when several reaction mechanisms coexist.

The article is organized as follows: in Section 2 we will review the classical GDF as the joint distribution of *θ* and *J*, followed by the definition of an intuitively simple QM quasi-probability joint distribution or QM GDF, *Q*_r_(*θ*,*J*), starting from the definition of the scattering amplitude. In Section 3 we will demonstrate the usefulness of the proposed *Q*_r_(*θ*,*J*) function to disentangle reaction mechanisms and to unveil quantum effects such as interference for three different systems and situations: inelastic collisions of Cl + H_2_; reactive D^+^ + H_2_ collisions; and H + D_2_ reactive scattering at a collision energy where quantum interference governs the shape of the DCSs for certain combinations of final and initial states. For all these systems, QM calculations have been carried out using the close-coupling hyperspherical method of Skouteris *et al.*,^19^ while quasiclassical trajectory (QCT) calculations have been performed using the procedure described in ref. 20. The reader not interested in the theoretical details can skip Section 2 and move directly to Section 3 where the potential of the GDFs is exemplified and discussed.

## 2 Theory

In this section the quasi-classical and quantum GDFs (or *J*–*θ* correlations) are presented. The detailed expressions to calculate them from the results obtained with QCT or from the QM scattering *S* matrix, respectively, are also given. As will be shown in the Results section, GDFs are a powerful tool to disentangle and describe reaction mechanisms, as they appear as distinct features in a *J*–*θ* representation. Moreover, no extra computational effort beyond that for the determination of the collision probability or differential cross section is required. The procedure to calculate GDFs is general and can be applied to all sorts of chemical reactions as long as the *S* matrix is obtained.

### 2.1 Classical generalized deflection function

The basis of the QCT method consists in calculating an ensemble of trajectories following a judicious sampling of the initial conditions to cover as much as possible the phase space relevant for the process to be studied, and complying with the state quantization of the reactants. The initial and final atom positions and linear momenta are then used to determine those initial and final properties (such as the angular momenta, scattering angle, final rovibrational states, *etc.*) necessary to characterize each individual trajectory. Finally, all is needed is to determine the average value of any conceivable property over the ensemble of trajectories. For example, the total reaction probability for a given value of the total angular momentum quantum number, *J*, discretely sampled can be obtained as:1
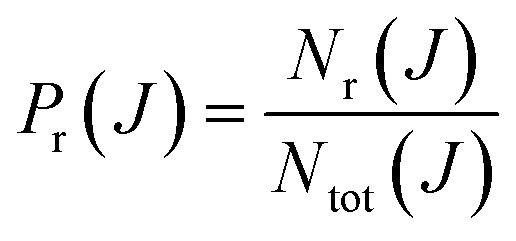
where *N*_r_(*J*) and *N*_tot_(*J*) are the number of reactive (or inelastic if that were the case) and total trajectories, respectively, for a given *J*. Recall that the total angular momentum ***J*** = ***𝓁*** + ***j***, where ***j*** is the rotational angular momentum and ***𝓁*** is the (relative) orbital angular momentum. We can define the corresponding quantum numbers, *J*, *𝓁* and *j*, such that |***J***| = [*J*(*J* + 1)]^1/2^*ℏ* and similarly for |***𝓁***| and |***j***|. These quantum numbers can be sampled continuously (real values) or discretely (integer values).

Eqn (1) is valid if the sampling in *J* is performed discretely and uniformly and, similarly, for the orbital angular momentum in the |*J* – *j*| ≤ *𝓁* ≤ *J* + *j* interval (for details see ref. 21). In addition, not all reactive trajectories need to have the same weight. Sometimes it is necessary to attribute different weights to each trajectory as is done in the Gaussian binning procedure^22^–^24^ to make the assignment of the final rovibrational states ‘more quantal’. In those cases, *N*_r_(*J*) in eqn (1) is replaced by *S*_w_, the sum of the weights of reactive (or inelastic) trajectories into a given final manifold of states. If one wishes to calculate a property that depends on more than one variable, for example *J* and *𝓁*, the scheme is the same except that now a joint probability has to be considered (say, the number of reactive trajectories with values of *J* and *𝓁*, *N*_r_(*J*,*𝓁*)).^21^ The aforementioned procedure is suitable for discrete variables, while for continuous variables it is a common practice to use histograms or, more elegantly, to fit the distributions to a series of orthogonal polynomials.^20^,^21^,^25^ Obviously, integration (or summation) over one of the variables of a given joint probability distribution leads to the probability distribution of the other variable. Moreover, if we split the original ensemble of trajectories into a series of sub-ensembles and calculate the respective joint probability distribution, it turns out that the global probability distribution can be easily recovered from the joint probability distributions for all the sub-ensembles; that is to say, the probability distributions are always additive. As we will see, this is not the case in QM scattering due to the coherences.

To illustrate the calculation of the classical correlation function, let us assume that the orbital angular momentum is sampled continuously in *𝓁* ∈ [0,*𝓁*_max_] with a weight of 2*𝓁* + 1, that is, the orbital angular momentum for the *i*-th trajectory is sampled as *𝓁*_*i*_(*𝓁*_*i*_ + 1) = *ξ*_*i*_[*𝓁*_max_(*𝓁*_max_ + 1)], where *ξ* is a random number in [0,1] (this is the same as sampling the impact parameter as *b* = *ξ*^1/2^*b*_max_).

We can conveniently define a *J*-partial cross section, *σ*_r_(*J*):2
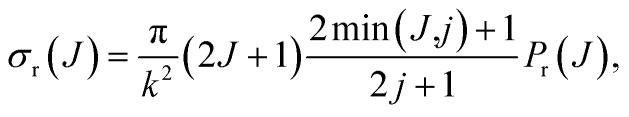
where *k*^2^ = 2*μ*(*E*_col_)/*ℏ*^2^ is the initial relative wavenumber vector, with *μ* being the atom-diatom reduced mass and *E*_col_ the collision energy. As defined, *σ*_r_(*J*) is nothing but a probability density function normalized such that its integral (or sum) over *J* is the integral cross section, *σ*_r_, either total or into a given final state.^21^ For discrete values of *J*, *σ*_r_(*J*) is usually denoted in the literature as *σ*_r_^*J*^.

The Monte Carlo normalized probability density function can be written as3
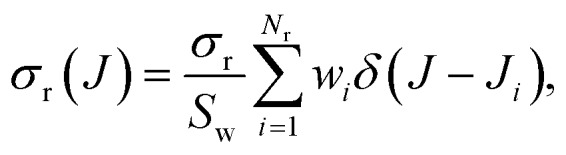
where *w*_*i*_ and *J*_*i*_ are the weight and the *J* value of the *i*-th trajectory. *S*_w_ is the sum of the weights of all the relevant reactive trajectories, *S*_w_ = ∑*w*_*i*_. In the simplest case, *w*_*i*_ would be a Boolean function whose value is one only for the specific reactive trajectories and zero otherwise, such that *S*_w_ = *N*_r_, the number of the considered reactive trajectories. As a convenient approximation, the Dirac delta functions can be replaced with a normalized Gaussian function4
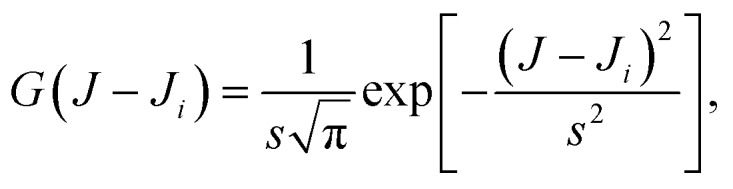
where the width, *s* = *Δ*_FWHM_/ln 2, is conveniently chosen depending on the average spacing of the successive values of *J*_*i*_ and the statistical uncertainty.

If the sampling in *J* (and in *𝓁*) is made continuous, the *J*-partial cross section can be expressed as an expansion in Legendre polynomials, *P*_*n*_(*x*):5
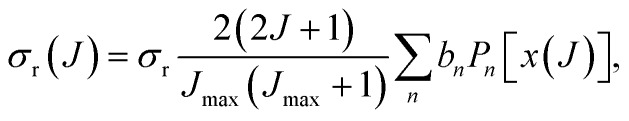
where *x* is a reduced variable, *x* ∈ [–1,1], given by6
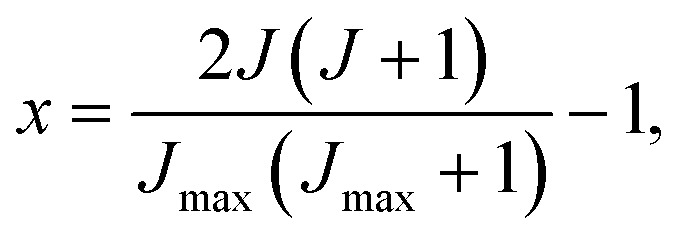
where *J*_max_ is the maximum value of the total angular momentum used in the calculation to ensure the convergence. The coefficients, *b*_*n*_, are given in terms of Legendre moments as7
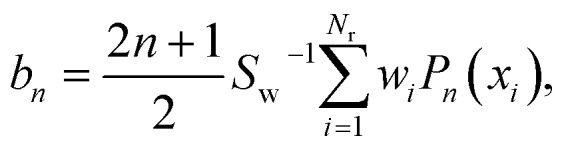
where *x*_*i*_ is the value of *x*, given by eqn (6), of the *i*-th trajectory, and *P*_*n*_(*x*) is the *n*-th order Legendre polynomial.

Similarly, the DCS can be expressed as an expansion in Legendre polynomials:8
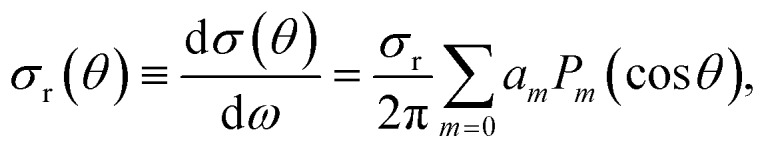
where *σ*_r_ is the integral cross section, and *a*_*n*_ represents the expansion coefficients whose values are given by:9
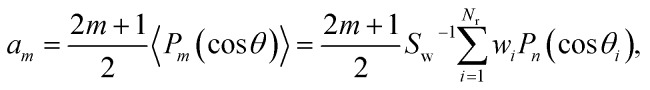
where where 〈*P*_*m*_(cos *θ*))〉 is the weighted average value of is the weighted average value of *P*_*m*_(cos *θ*) over the ensemble of the relevant trajectories.

The joint probability distribution of *J* and *θ*, already used in ref. 15 and 26, normalized to the integral cross section, can now be expressed as a double expansion in Legendre polynomials10
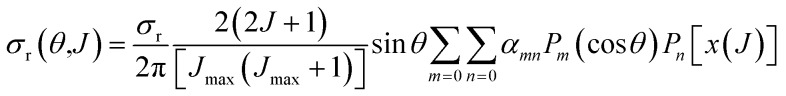
where the coefficients *α*_*mn*_ are given by:11
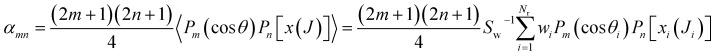



The Monte Carlo expression of the deflection function can be expressed as a sum of Gaussian functions given by12
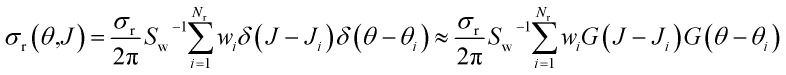
where *J*_*i*_ and *θ*_*i*_ represent the values of *J* and *θ* for the *i*-th trajectory. *G*(*J* – *J*_*i*_) and *G*(*θ* – *θ*_*i*_) denote normalized Gaussian functions with width parameters *s*_*J*_ and *s*_*θ*_, centred in *J*_*i*_ and *θ*_*i*_, respectively.

Integration of eqn (10) or (12) over *θ* and the azimuthal angle provides the *J*-partial cross section of eqn (5) and (3). Alternatively, integration over *J* in these equations gives *σ*_r_(*θ*)sin *θ*. Hereinafter we will indifferently denote *σ*_r_(*θ*,*J*) as the QCT generalized deflection function (QCT GDF) or the QCT *J*–*θ* correlation function.

### 2.2 QM generalised deflection function: QM *J*–*θ* correlation function

Due to their classical nature, there is no restriction in QCT calculations to obtain any correlation between two or more properties. After all, each trajectory is characterized by specific values of every initial or final property. However, this is not the case for QM scattering calculations, which makes the analysis based on pure QM calculations not so trivial. From the QM scattering calculations we only obtain as an outcome the scattering matrix (*S*-matrix) that relates the initial states of the reactants and the final states of the products. This means that to obtain a dynamical observable from a QM calculation, we need a recipe to extract its value from the elements of the *S*-matrix. For the particular case of closed shell diatomic molecules in the helicity representation (body-fixed frame), and a given value of *J*, they are characterized by three quantum numbers for each arrangement: *v* and *j* (*v*′ and *j*′) that define the vibrational and rotational states respectively, and the helicity *Ω* (*Ω*′), the projection of *j* (*j*′) (or *J*) in the approach (or recoil) direction.

Some observables can be readily extracted from the *S*-matrix. This is the case of *P*_r_(*J*) that, for a given initial state and total energy, can be calculated as follows:13
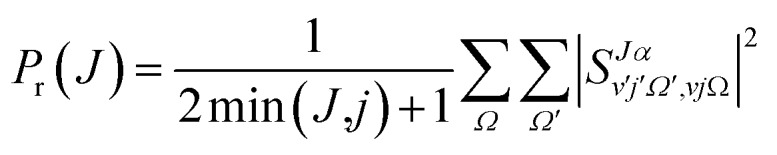
where the sum runs over the desired product states (or, if referred to state-to-state, without summing over *v*′ and *j*′). Hereinafter, subscripts for *v*, *j*, *v*′, and *j*′, and the chemical arrangement will be omitted for clarity. The integral cross section can be written in terms of the reaction probabilities as14
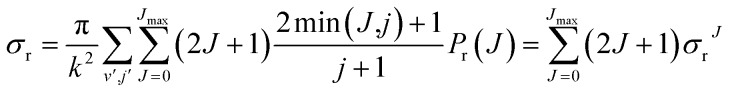
where *J*_max_ is the maximum value of *J* necessary for convergence. *σ*_r_^*J*^ is the *j*-partial cross section already mentioned in the previous subsection.

The derivation of vector properties such as the DCS from the *S*-matrix is not so straightforward, firstly, because we need to include the angular dependence, and secondly, because they involve coherences between different elements of the *S*-matrix. It is convenient to express the DCS in terms of the scattering amplitudes, which are defined as:15
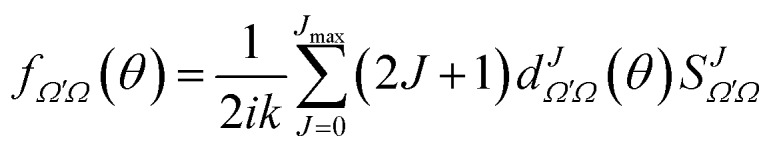
where *d*_*Ω*′*Ω*_^*J*^(*θ*) is the Wigner *d*-matrix. The DCS can now be written using the scattering amplitudes as:16
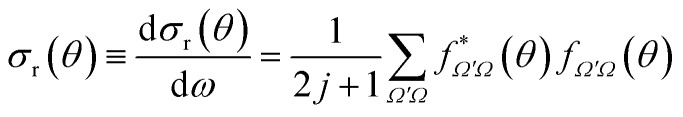



From eqn (15) and (16) it is clear that the DCSs for state-to-state processes are additive, even when they are resolved in *Ω*′ and *Ω*. However, the squaring of the sum over *J* in eqn (15) makes the DCS no longer additive in *J*, *i.e.*, there are coherences (cross terms) between different *J* partial waves. This property is a reflection of the wave nature of quantum mechanics, so that two or more “paths” (impact parameters or *J*) leading to scattering at the same angle will interfere. Hence, in principle, it is not possible to separate the contribution of two partial waves to the converged DCS. It is worth noticing that usually coherences are only important between nearby values of *J*^27^ so, for certain cases, it is possible to extract the contributions from one or many mechanisms from the DCS.

To calculate a QM *J*–*θ* correlation function we would need to extract the contribution of each *J* to the total DCS. Furthermore, for a QM correlation function to be reliable it should be additive so that the sum over *J* leads to the converged (summed over all *J*-partial waves) DCS. In principle, one could compute it by neglecting all coherences between different *J*s. This would be equivalent to using the random phase approximation that lies in the core of the statistical model,^28^,^29^ giving rise to forward–backward symmetric DCSs. For non-statistical (direct) reactions, a symmetric DCS is in clear disagreement with the experimental and QM results, and hence neglecting coherences can be considered as a very inappropriate approximation to obtain a QM correlation function. Instead, to devise a QM GDF, we will start by defining a *J*-partial dependent scattering amplitude as:17
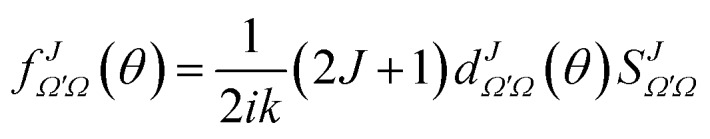
where |*Ω*|, |*Ω*′| ≤ *J*. The (total) scattering amplitude can now be written as18
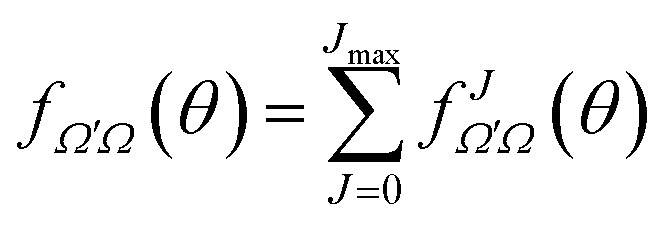



The DCS can be expressed as a function of the *J*-partial scattering amplitudes:19
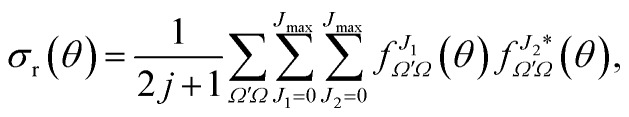
which is the same as eqn (16). Without any approximation, eqn (19) can be rearranged to20
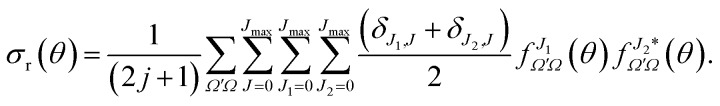



Eqn (19) and (20) only differ in the presence of an additional sum over *J* in eqn (20) that is compensated with the term (*δ*_*J*_1_,*J*_ + *δ*_*J*_2_,*J*_)/2, which guarantees that both equations include the same number of cross products and hence that they are equivalent. The advantage of eqn (20) is the presence of a separate summation over *J* that allows us to define a function that depends on a single *J* and *θ*; that is, a quantum analog to the classical joint probability distribution, and we will denote that as *Q*_r_(*θ*,*J*),21
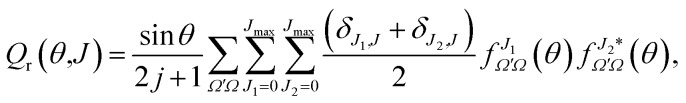
where sin *θ* has been added so the sum over *J* and integration over *θ* recovers *σ*_r_. To help the interpretation of the quantum correlation function defined in this work, eqn (21) can be recast as22
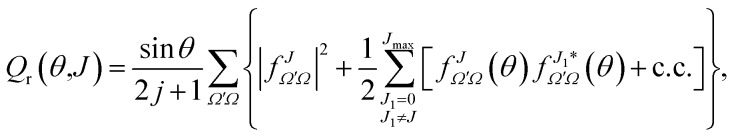
where c.c. stands for the respective conjugate complex. Eqn (22) contains the square of the *J*-dependent scattering amplitude,|*fJΩ′Ω*(*θ*)|^2^, plus a halved summation of *J*_max_ terms over all the total angular momenta *J*_1_ ≠ *J*, which are the coherent terms. The factor 1/2 can be readily explained. A half of the summation corresponds to a given *J*. The other half will appear in the *Q*_r_(*θ*,*J*′) expression of a previous or subsequent value, *J*′ ≠ *J*, such that summation over *J* will provide the DCS. Otherwise we would be counting twice the cross terms.

In the absence of coherences, that is, in the random phase approximation limit, the only surviving term would be that depending on *J* only. The remaining terms account for the possible interference that most of the time can be expected to be only important between partial waves in a restricted range of *J* in [*J* – Δ*J*, *J* + Δ*J*].^15^,^16^ However, as will be shown below, interference can also take place between partial waves that cover the full range of angular momentum leading to scattering.

The QM *J*–*θ* correlation function (or QM GDF) shares some important properties in common with its classical counterpart, *σ*_r_(*θ*,*J*). As in the classical case, summing eqn (21) over *J* leads to the DCS given by eqn (20) multiplied by sin *θ*, *σ*_r_(*θ*)sin *θ*. Similarly, integration over the scattering angle and the azimuthal angle23
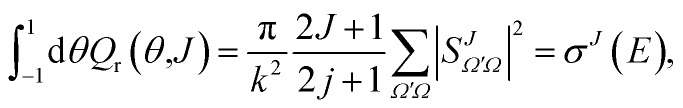
gives the *J*-partial cross section, eqn (14), as in the classical treatment.

In spite of the similarities between the classical *σ*_r_(*θ*,*J*) (eqn (10) or (12)) and the quantum *Q*_r_(*θ*,*J*) (eqn (21)), there are important differences between them. The latter is not a true joint probability distribution (and, hence, a GDF in the classical sense) since it includes coherences between different values of *J*. Moreover, it can take negative values whenever there is destructive interference between pairs of *J* values, although when summed over *J* up to *J*_max_, the GDF is always positive. Notwithstanding the differences, as will be shown in Section 3, when the interference is not significant, classical and quantum correlation functions bear a close resemblance.

It is sometimes useful to calculate the angular distributions for a subset of partial waves. These angular distributions, labeled as DCS([*J*_*i*_,*J*_*k*_]), can be calculated by restricting the sum over *J* in eqn (20) to a given range of *J*, *J* ∈ [*J*_*i*_,*J*_*k*_],24
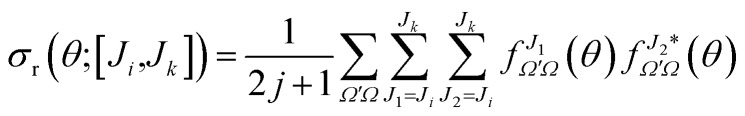



The partially summed QM DCS, DCS([*J*_*i*_,*J*_*k*_]), includes all coherences between partial waves within the [*J*_*i*_,*J*_*k*_] range but none outside this range. In addition, like the DCS itself, DCSs([*J*_*i*_,*J*_*k*_]) are not additive, especially if there is interference between different groups of *J*s. This is again in contrast to the corresponding classical partial cross section summed over the [*J*_*i*_,*J*_*k*_] interval.

It is also possible to define a deflection function by restricting the sum over a given [*J*_*i*_,*J*_*k*_] range of *J*, *Q*_r_(*θ*;[*J*_*i*_,*J*_*k*_]), as25
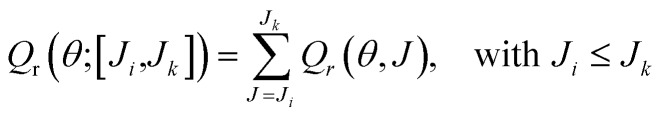



In spite of the similarities between the partial DCS([*J*_*i*_,*J*_*k*_]) and *Q*_r_(*θ*;[*J*_*i*_,*J*_*k*_]) (and the fact that in the limit of the complete convergence interval, *J*_*i*_ = 0 and *J*_*k*_ = *J*_max_, both functions are identical), there are two main differences between them: (i) the latter also includes coherences between partial waves outside the [*J*_*i*_,*J*_*k*_] range so it may take negative values (if destructive interference prevails for some scattering angles); (ii) the *Q*_r_(*θ*;[*J*_*i*_,*J*_*k*_]) values for different intervals defined in eqn (25) are additive as in the classical case. Hence, from the comparison between the partially summed DCSs and partial QM correlation function, it is straightforward to ascertain if interference phenomena arise and the partial waves that contribute to them. It must be noted that in the classical case both functions are the same.

### 2.3 Quantum deflection functions (QDFs)

The idea of a semiclassical deflection function was first developed by Ford and Wheeler in the context of elastic scattering using the stationary phase approximation,^8^ and later consolidated by Bernstein.^9^ The semiclassical approximation techniques proved to be very useful to gain insight into the physical nature of scattering, making it possible to extract qualitative inferences and easing the interpretation of the quantum results.^9^,^30^–^34^

The semiclassical deflection function, *Θ*(*𝓁*_*θ*_), is related to the phase shift, *η*_*𝓁*_, by26
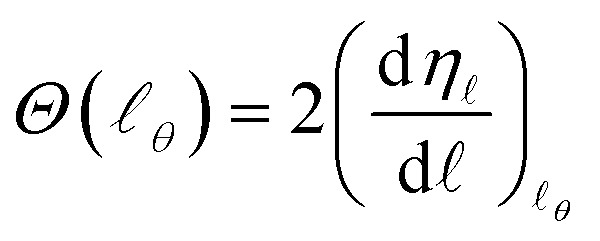
where *Θ* = ±*θ* for repulsive and attractive potentials, respectively, and the derivative of *η*_*𝓁*_ is evaluated at *𝓁*_*θ*_, the *𝓁*-value of the stationary phase. The phase shift can be written in terms of the *S* matrix as

27 *S*_*𝓁*_ = e^2*iη*_*𝓁*_^

Hence,28
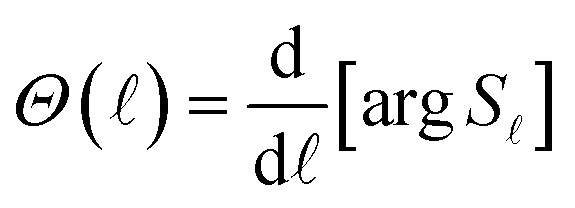



Eqn (26) also holds if the exact-QM phase shifts are defined, leading to a quantum mechanical DF that can be applied for elastic scattering processes even for soft edge potentials.^35^

In a series of articles, Connor and co-workers extended the semiclassical treatment and developed a quantal version of the deflection function applicable to the most general case of inelastic and reactive scattering.^10^–^12^ It is thus pertinent to compare our proposed GDF with the QDF devised by Connor and coworkers. In what follows, we will briefly summarize the main equations of that method for our present purposes.^10^

For given initial and final rovibrational states the QDF, denoted as *Θ˜_Ω′Ω_*, is defined as29
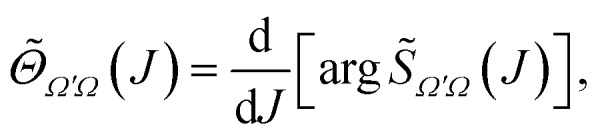
where *SS̃*_*Ω*′*Ω*_(*J*) represents the modified scattering matrix elements that can be calculated directly from the scattering matrix:

30 *SS̃*_*Ω*′*Ω*_(*J*) = exp(*i*π*J*)*SJΩ*′*Ω*

It should be highlighted that arg *SS̃*_*Ω*′*Ω*_(*J*) does not denote the principal value, but it is defined as a continuous function as follows:31
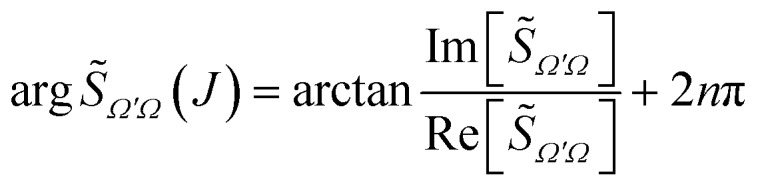
where *n* is a positive or negative integer number, whose value is arbitrarily set to 0 for *J* = 0, and for *J* > 0 is selected such that arg *SS̃*_*Ω*′*Ω*_(*J*) – arg *SS̃*_*Ω*′*Ω*_(*J* – 1) < π is a continuous function.

The differences between the QDF and *Q*_r_ need to be emphasised. Whilst the latter is a sort of probability density function in terms of both *θ* and *J*, which appears as a 3D plot of the scattering intensity as a function of both *J* and *θ* containing information about the scattering intensity and the presence of constructive or destructive interference, the QDF represents a one-dimensional relationship between the deflection angle (or the scattering angle) and the angular momentum *J*. Moreover, as shown in the previous subsection, if *Q*_r_(*θ*,*J*) is summed over *J*, one gets the DCS, in contrast to the QDF. Another difference is that whilst the QDF is defined for each pair of *Ω* and *Ω*′ values, the GDF defined in this work can include the average over the reactant's and the summation over product's helicities as shown in eqn (21), although it can also be calculated for specific values of *Ω* and *Ω*′, as will be shown below. Apart from these differences, one would expect a confluence with regard to the relationship between the scattering angle and angular momentum, at least if each partial wave can be mostly associated with one range of scattering angles.

## 3 Results and discussion

In this section we will examine three case studies to illustrate the usefulness of the QM generalized deflection function. First of all, we will study the inelastic collisions of Cl + H_2_, where the QCT deflection function succeeded in explaining the quantum results. Next, we will study the reactive D^+^ + H_2_ system, a prototype of barrierless reactions, where we expect no correlation between *J* and *θ*. Finally, we will apply the QM GDF to reactive scattering between H and D_2_ at high collision energies where quantum interference governs the angular distributions for certain combinations of final and initial states. In all cases, the main goal will be to exploit the capabilities of the QM GDF to reveal the existence of competing mechanisms and interference between them.

### 3.1 Inelastic collisions between Cl and H_2_

The first example in which we will use the GDF proposed in this work is the inelastic collisions between Cl and H_2_(*v* = 0, *j* = 0). This system has been extensively studied both computationally and experimentally.^36^–^40^

Regarding inelastic collisions, some interesting features emerged from previous studies.^41^,^42^ QM and QCT calculations using the BW2 PES^43^ showed that at relatively high collision energies (*E*_coll_ > 0.6 eV) and for small Δ*j* values (Δ*j* = *j*′ – *j*), the inelastic probabilities, *P*_r_(*J*), exhibit two maxima separated by a minimum in the QCT and QM results. This minimum was identified as that corresponding to the glory impact parameter. The analysis of the results showed that there are two mechanisms responsible for the inelastic scattering, possibly associated with different regions of the PES and resulting in very different stereodynamical behaviours.^41^,^42^ Both dynamical regimes depend primarily on the value of the total (here also orbital) angular momentum: (i) for *J*s below the glory impact parameter, collisions seem to take place following a sort of “tug-of-war” mechanism,^44^ which indicates the stretching of the H–H bond;^42^ and (ii) for *J* ⪆ 40 collisions can be assigned to rainbow scattering in which the attractive part of the PES is sampled.^41^ For transitions involving higher Δ*j*, which require more head-on collisions, the contribution of high impact parameters wanes rapidly, and the second maximum in the *P*_r_(*J*) leading to small scattering angles disappears. The semi-quantitative agreement between the classical and quantum *P*_r_(*J*) and DCSs seems to indicate that quantum effects associated with interference between the two groups of partial waves are not expected to be important.^41^ Therefore, the Cl + H_2_(*v* = 0, *j* = 0) inelastic scattering seems to be a good example of a collision system in which the QCT and QCT GDFs would be similar.

**Fig. 1:**
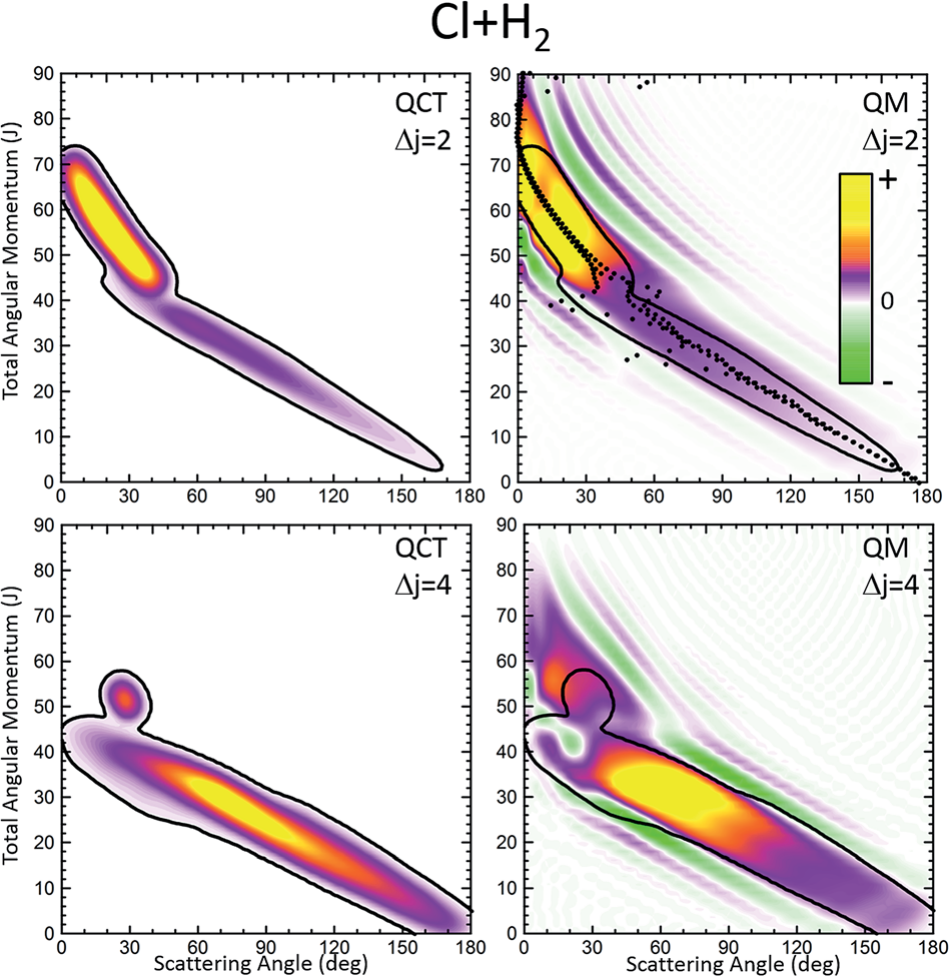
Comparison of the QCT deflection functions (left panels) and the QM *J*–*θ* correlation or GDF (right panels) for the Cl + H_2_(*v* = 0, *j* = 0) → Cl + H_2_(*v*′ = 0, *j*′ = 2, 4) inelastic collisions at *E*_col_ = 0.73 eV. Top panels, Δ*j* = 2; bottom panels, Δ*j* = 4. The contour of the QCT *J*–*θ* correlation function has been added to the QM *Q*_r_ to ease the comparison. The green colour corresponds to negative values and hence destructive interference (*Q*_r_(*θ*,*J*) < 0). For comparison purposes, the QDF is shown on top of the QM GDF for Δ*j* = 2 using black dots.

Fig. 1 displays the QCT and the QM deflection functions for the *j* = 0 → *j*′ = 2 and *j* = 0 → *j* = 4 transitions (top and bottom panels, respectively) at *E*_coll_ = 0.73 eV. The left panels show the QCT *σ*(*θ*,*J*). The two different dynamical regimes can be easily distinguished. For Δ*j* = 2, the high-*J* mechanism is preeminent and gives rise to scattering into *θ* < 50°. The low-*J* mechanism appears in the deflection function as a narrow band that extends from *θ* = 40° to *θ* = 180° and comprises *J* values from 0 to 40. The negative slope, common to both regimes (although with different values), is characteristic of direct collisions, and follows the simple correlation of low (high) impact parameters leading to high (small) scattering angles. For Δ*j* = 4, the prevailing mechanism is that corresponding to *J* ≤ 40 values, and the high-*J* mechanism appears as a small island in the *J*–*θ* map, centered at *J* = 50 and *θ* = 30°.

The equivalent QM *Q*_r_(*θ*,*J*)'s, shown in the right panels of Fig. 1, bear close similarities to their classical counterparts, although with some noticeable differences. For Δ*j* = 2, the high-*J* mechanism, responsible for most of the scattering, extends to larger values of *J*, it is broader, and it is flanked by a series of stripes, some of negative value (green colour) associated with destructive interference. The negative slope of the low-*J* mechanism is also observed, although in this case both mechanisms merge at *J* ∼ 45. There are also a series of negative stripes parallel to the main band which cause a small decrease of the DCS. It should be noticed that, for the sake of clarity in the figure, the QM GDF has been smoothed given the discrete character of *J*. The same procedure will be followed for all remaining 3D plots of this article. For Δ*j* = 4, the QM-DF also extends to larger *J* values and the high-*J* mechanism covers a broader *J*–*θ* region than in the QCT case. As in the classical case, for this transition, the low-*J* mechanism bears away most of the scattering.

**Fig. 2:**
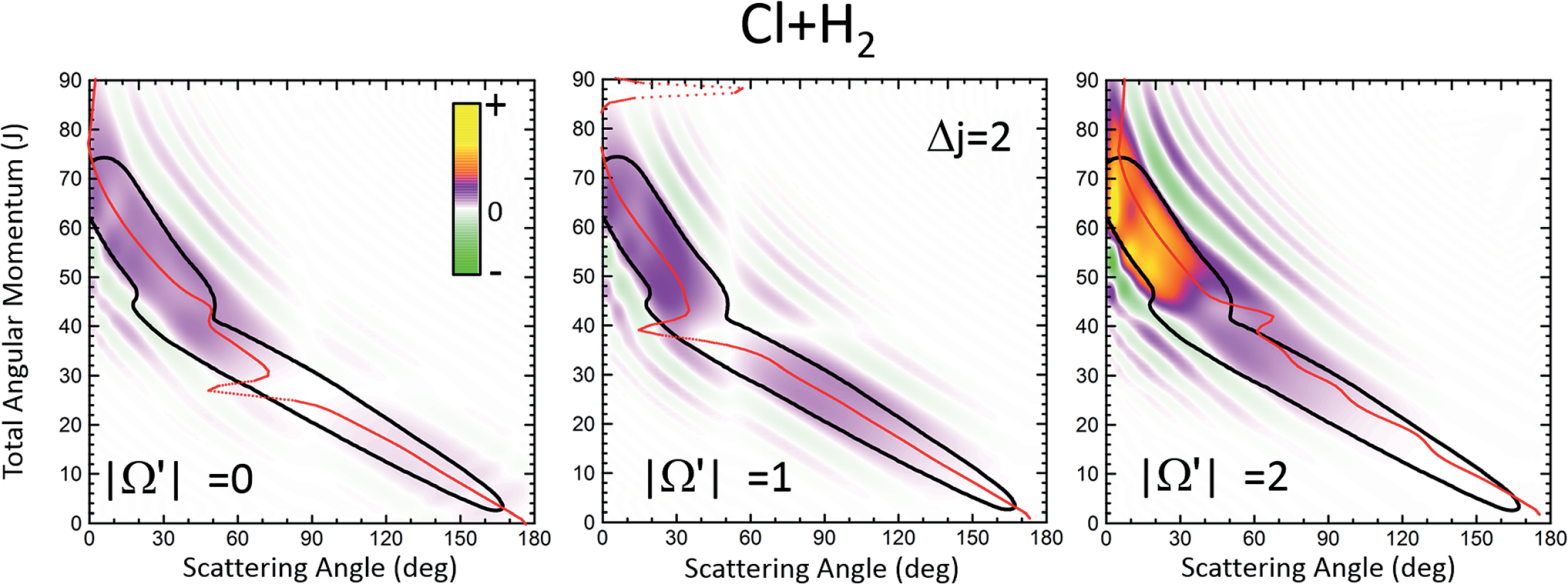
QM *J*–*θ* correlation function at *E*_col_ = 0.73 eV for the Cl + H_2_(*v* = 0, *j* = 0) → Cl + H_2_(*v*′ = 0, *j*′ = 2, |*Ω*′| = 0, 1, 2) inelastic collisions resolved in *Ω*′ helicity states. The corresponding QDFs devised by Connor and coworkers are also shown using solid red lines.

The results of the QDF for Δ*j* = 2 are also shown as a dotted lines along with the present *Q*_r_(*θ*,*J*). The points corresponding to *Ω*′ = 0, 1 and 2 are all included. As can be seen, the QDF follows almost exactly the middle line (reproducing the two different slopes) of the present QM GDF and is also in good agreement with the corresponding QCT function. More detailed information is shown in Fig. 2, where the *Q*_r_(*θ*,*J*,*Ω*′) is plotted separately for each of the three possible *Ω*′ values along with the corresponding QDF. As can be seen, the agreement is excellent and the QDF matches almost exactly the most probable dependence of *θ* with *J* found with the present *Q*_r_(*θ*,*J*). It should be pointed out, however, that the latter also carries information on the intensity of scattering for each *J*–*θ* region, and about the presence of constructive and destructive interference. Indeed, the information conveyed by the present *Q*_r_(*θ*,*J*,*Ω*′) goes well beyond that obtained by the QDF. As can be seen, most of the intensity of the high-*J* mechanism corresponds to *Ω*′ = 2, indicating that the product's *j*′ rotational angular momentum lies preferentially along the recoil velocity, whilst that corresponding to low-*J* is more isotropic with some preference for *Ω*′ = 1.^42^

**Fig. 3:**
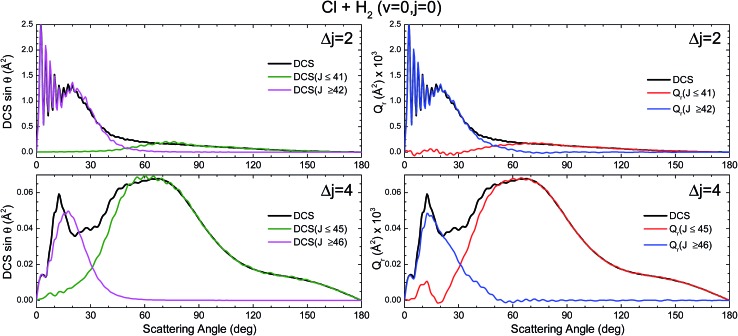
Comparison of the DCS partially summed over the indicated *J* interval, DCS([*J*_*i*_,*J*_*k*_]) (defined in eqn (24)) (left panels) and the QM deflection functions summed over the same *J* interval *Q*_r_(*θ*;[*J*_*i*_,*J*_*k*_]) (defined in eqn (25)) (right panels) for the inelastic collisions between Cl and H_2_(*v* = 0, *j* = 0) at *E*_col_ = 0.73 eV and Δ*j* = 2 (top panels) and Δ*j* = 4 (bottom panels).

The partial DCS, eqn (24), and the QM *Q*_r_(*θ*,Δ*J*) summed over the indicated range of *J*, eqn (25), are shown in the left and right panels of Fig. 3, respectively, for Δ*j* = 2 and 4. The two *J* intervals have been chosen to comprise partial waves corresponding to low-*J* (*J* ≤ 41 for Δ*j* = 2 and *J* ≤ 45 for Δ*j* = 4) and high-*J* (*J* > 41 for Δ*j* = 2 and *J* > 45 for Δ*j* = 4). Therefore, the two magnitudes are broken down in their contributions from the two intervals for comparison purposes. It should be recalled that if the whole range of *J* is included, both magnitudes become identical, corresponding to the converged (including all partial waves) DCS. However, whilst the partial DCS only encompasses those coherences within the chosen interval, the partially summed QM GDF comprises all possible coherences internal and external to that interval.

The first consideration to be held is the similarity of the respective decompositions of the partial DCSs and the summed QM GDFs *Q*_r_(*θ*;Δ*J*), of the left and right panels. As a second consideration, for Δ*j* = 2, the incoherent sum of *σ*(*θ*;*J* ≤ 42) and *σ*(*θ*;*J* > 42) reproduces fairly well the converged DCS (recall that the partial DCSs are not additive), evincing that interference between the two mechanisms is practically negligible. A similar analysis was performed in ref. 42 leading to the same conclusion. This is further confirmed by inspection of *Q*_r_(*θ*;Δ*J*), shown in the right-top panel, which is almost identical to the partial DCSs, except for a few differences in the forward region. For the case of Δ*j* = 4 the situation is much the same as that for Δ*j* = 2. The only main difference between partial DCSs and *Q*_r_(*θ*;Δ*J*) can be observed at forward scattering angles *θ* = 10–30°. As can be seen, there is a peak centred at *θ* = 12° in the *Q*_r_(*θ*;*J* < 46) which is absent in the respective *σ*_r_(*θ*,*J* < 46). This implies that there are some, relatively unimportant, interference between the two groups of partial waves. Returning to Fig. 1, it is possible to associate this effect with the feature that appears with a ‘hook’ at the top corner of the right-bottom panel of that figure.

It must be pointed out that the above discussion does not imply that for Δ*j* = 2 there is no interference within one of those groups of partial waves. By the inspection of the right-bottom panel of Fig. 1, it is obvious that in the forward region and at high *J* > 40 there is high constructive and destructive interference that is the origin of the oscillations observed at *θ* < 30° in Fig. 3.

### 3.2 Reactions that go through a long-lived complex, D^+^ + H_2_

A contrasting system is the D^+^ + H_2_ → HD + H^+^ reaction on its first 1^1^A′ adiabatic PES. As is well known, the PES is barrierless and rather featureless, overwhelmingly dominated by a very deep well of 4 eV from the asymptotes.^45^,^46^ Given its importance in astrochemistry, it has been extensively studied both theoretically and experimentally (see, for example, ref. 47–57 and references therein).

**Fig. 4:**
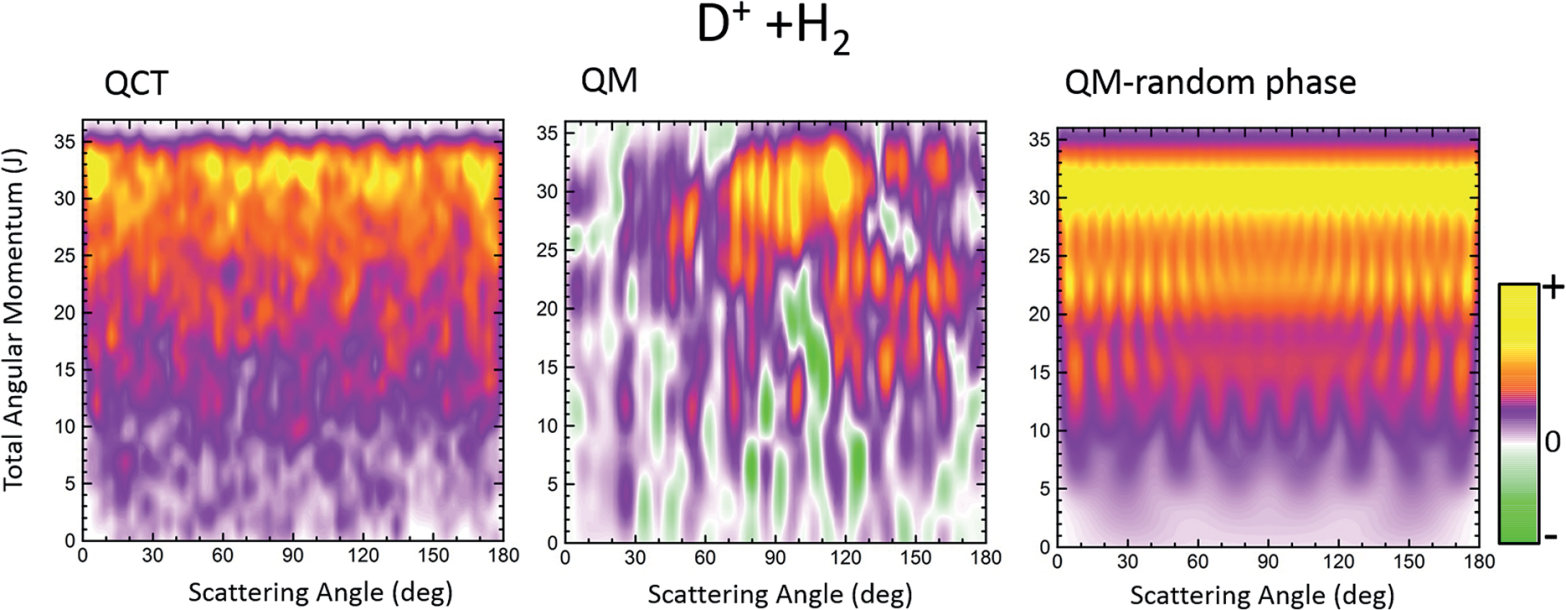
Comparison of the QCT GDF and its QM analogue for the D^+^ + H_2_ reaction at *E*_col_ = 0.15 eV. The rightmost panel depicts the QM GDF corresponding to the random phase approximation. The results are for HD(*v*′ = 0, *j*′ = 1).

We will focus on the results at a sufficiently low energy, *E*_coll_ = 150 meV and HD(*v*′ = 0, *j*′ = 1) formation, where the statistical (ergodic) assumption seems to hold.^52^–^55^ Indeed, at this energy, the D^+^ + H_2_ reaction proceeds through the formation of a long-lived complex, and the shape of *P*_r_(*J*) and the product state distributions follow the trend predicted by statistical methods.^55^ Hence, this seems to be a good example to test the reliability of the *Q*_r_(*θ*,*J*) in statistical reactions. In Fig. 4 three GDFs are shown: the classical function, the QM *Q*_r_(*θ*,*J*) and the quantum one under the assumption of the random phase approximation. The latter implies that there are no correlations between different *J*s, so that it only includes the |*fJΩ′Ω*(*θ*)|^2^ terms in eqn (22). In all three cases, as expected for a statistical reaction, there is no clear correlation between *J* and *θ*: all *J*s seem to contribute to every scattering angle. The only remarkable feature in the classical *J*–*θ* correlation function is the largest probabilities found at high *J*, due to the fact that the *P*_r_(*J*) is flat until it decreases abruptly when reaching *J*_max_. The *Q*_r_(*θ*,*J*), shown in the middle panel of Fig. 4, indicates the presence of high destructive (green) and constructive (red/yellow) interference that will give rise to multiple oscillations in the DCS over the whole range of scattering angles. However, coherences even if they occurred between partial waves with separated *J* values are so numerous that their effect is smoothed out to some extent. This is the basic assumption in the random phase approximation,^29^,^58^ which allows one to calculate coarse-grained product's state distribution DCSs and other vector correlations^59^ by neglecting the coherences between different total angular momenta. The right panel of Fig. 4 shows the random phase approximated DF, where all the coherences have been neglected by only keeping the diagonal terms. Apart from the discrete character of *J*, the similarity with the QCT GDF is remarkable. For this reaction, the QDF results in a highly oscillating function due to the superposition of nearside and farside scattering.^60^

**Fig. 5:**
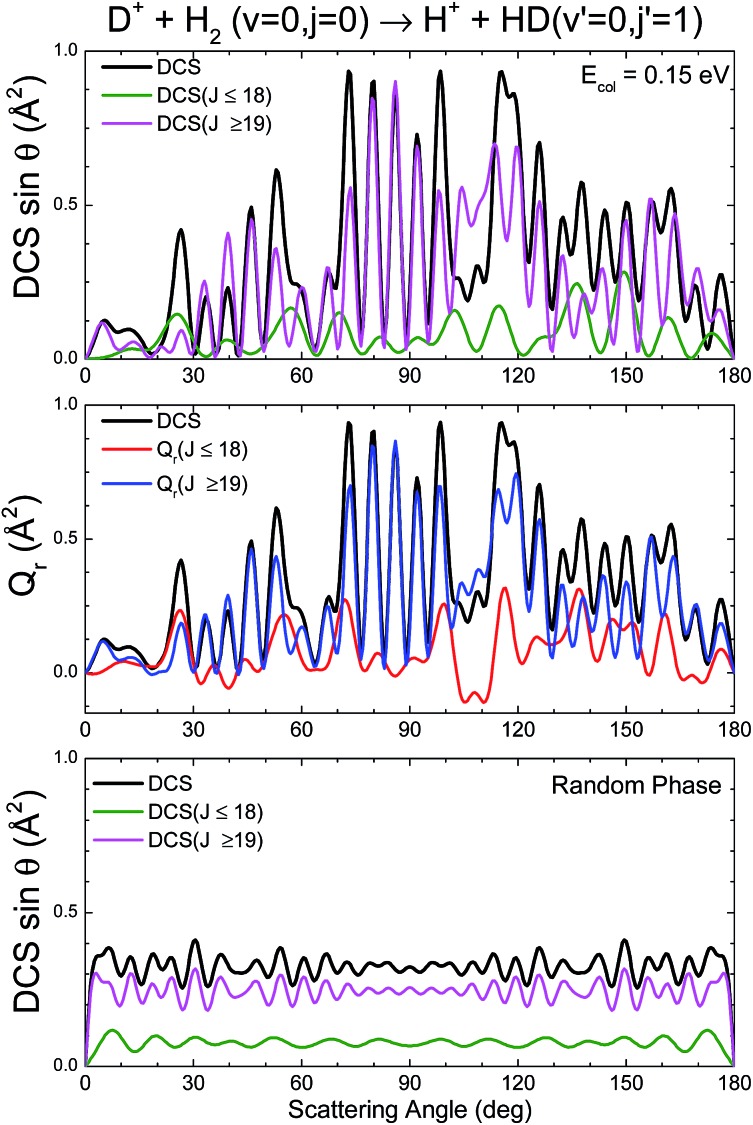
Comparison of the *Q*_r_(*J*,*θ*) and the DCS for the D^+^ + H_2_ → HD(*v*′ = 0, *j*′ = 1) + H^+^ reaction at *E*_col_ = 0.15 eV.

The partial and total DCSs, as well as the *Q*_r_(*θ*,[*J*_*i*_,*J*_*k*_]) summed over limited ranges of *J*, are shown in the top and middle panels, respectively, of Fig. 5. The *J* dividing value between low-*J* and high-*J* values has been chosen somewhat arbitrarily as *J*_max_/2, since no hint of change of mechanism seems to be appreciable in either the QCT or the QM GDF. As expected from the QM GDF, the DCSs with the full QM calculation exhibit many oscillations in the whole range of scattering angles, reflecting the high interference that is apparent in Fig. 4. The partial DCSs and their respective *Q*_r_(*θ*,Δ*J*) summed in [0,18] and [19,35] are fairly similar with some interesting differences. Specifically, the inspection of the partially summed *Q*_r_(*θ*,Δ*J*) makes it possible to identify the dip in the converged DCS at 100–110° as a result of destructive interference between the partial waves with Δ*J* ≤ 18 (negative value) and Δ*J* > 18, information that cannot be extracted from the partially summed DCS, that only includes interference within each interval. This destructive interference can be also seen in the QM GDF shown in the middle panel of Fig. 4 as the green stripes (negative value) at those angles.

The partial DCSs, which under the random phase approximation coincide with the *Q*_r_(*θ*,Δ*J*), are shown in the bottom panel of Fig. 5. There are still some oscillations that are basically the result of the summation of reduced rotation matrix terms, [*dJΩΩ′*(*θ*)]^2^ (eqn (17)). The resulting random phase DCSs are strictly symmetric, peaking at forward and backward angles (recall that the represented DCSs have been multiplied by sin *θ*). Although at first glance there seems to be a poor approximation to the actual DCSs, it must be borne in mind that the observed oscillations change rapidly with the collision energy and initial states, and hence they would be barely discernible under experimental conditions.

### 3.3 Direct reactions: H + D_2_

The third system we will be concerned with is the H + D_2_ reaction, possibly the most extensively studied reaction, and indeed the benchmark system in reaction dynamics. Although from many points of view it can be considered as the simplest reaction, its dynamics is far richer than it could be expected;^13^,^14^,^61^ indeed, when investigated in detail it still provides unexpected results.^15^,^62^,^63^ Very recently, the angular distributions of state resolved HD formed in collisions between H and D_2_ at *E*_col_ = 1.97 eV were measured using the photoloc technique.^7^ For HD(*v*′ = 1, low *j*′) states the angular distributions in the backward hemisphere were dominated by a series of peaks and dips whose origin was traced to interference between the two mechanisms described in ref. 14 and 15. For both, higher *v*′ and/or *j*′ rovibrational states, one of the mechanisms disappears and so does the interference pattern in the DCS. In previous studies it was shown that the QCT GDF was crucial for the right interpretation and assignment of the observed interference pattern.^15^,^16^ It can thus be expected that the QM *Q*_r_(*θ*,*J*) will convey at least the same and presumably even more information about the reaction mechanism. Therefore, the state resolved H + D_2_ reaction would be an excellent system to assess the quantum analogue to the classical GDF as we can test its performance under three different scenarios: (i) HD(*v*′ = 1, *j*′ = 0) formation, where the interference pattern is conspicuous and dominates the shape of the DCS in the backward hemisphere; (ii) higher *j*′, for instance HD(*v*′ = 1, *j*′ = 5), where oscillations start to disappear; (iii) higher *v*′, *i.e.*, HD(*v*′ = 3, *j*′ = 0), where no clear oscillations were observed in backward scattering. In what follows, we will show the QM GDF, partial DCS and the *Q*(*θ*,Δ*J*) summed over the appropriate ranges of *J* for these three different scenarios. All calculations were carried out on the BKMP2 PES.^64^

**Fig. 6:**
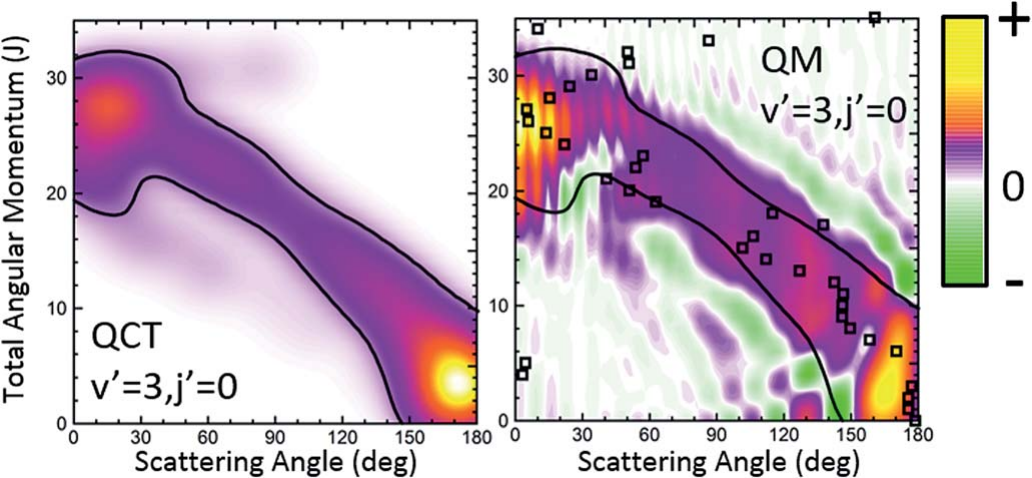
QCT and QM generalized DFs for the H + D_2_ → HD(*v*′ = 3, *j*′ = 0) + D reaction at *E*_col_ = 1.97 eV. The contour of the classical generalized DF has been added to the plot representing the QM *Q*_r_(*θ*,*J*) to highlight the similarities and differences. The open squares represent the QDF.

Let us first turn our attention to those collisions leading to HD(*v*′ = 3, *j*′ = 0) whose QCT and QM GDFs are depicted in Fig. 6. The QCT *σ*_r_(*θ*,*J*) shows the typical profile of a direct reaction mechanism, similar to that observed for the inelastic collisions between Cl and H_2_, that is, a band running diagonally across the *θ*–*J* map with low *J* giving rise to backward scattering and high *J* correlating with forward scattering. In this case, the mechanism covers the whole range of scattering angles with one maximum in the forward and another in the backward region. Moreover, there seems to be no other mechanism to compete with it. Not surprisingly, QCT and QM GDFs are very similar, showing the same structure moving from backwards to forwards. However, although the QM results were somewhat smoothed out for the sake of clarity, we can still observe a series of constructive and destructive interference manifested as stripes, especially in the forward scattering region. In addition, the main band is flanked by two small green stripes (destructive interference) that will give rise to oscillations in the DCS.

**Fig. 7:**
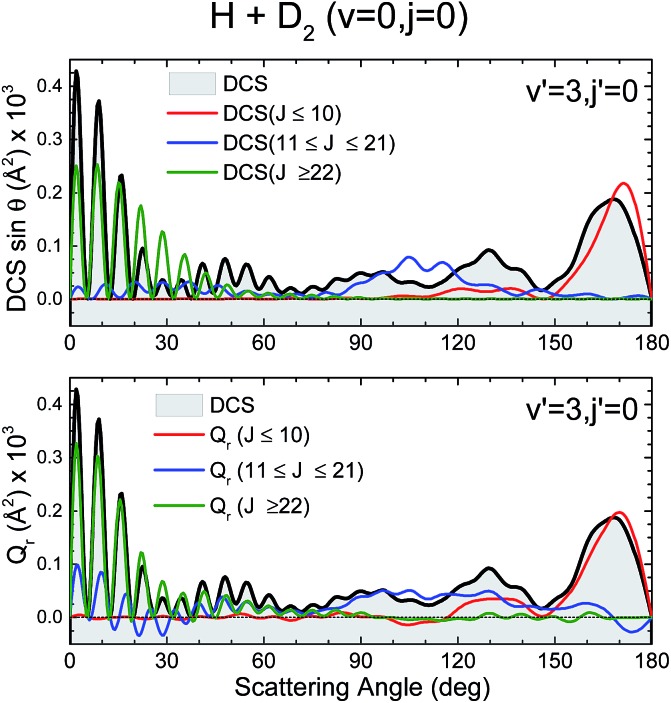
Comparison of the partial DCS (upper panel) and the QM *Q*(*θ*,Δ*J*) summed over the same *J* contributions (bottom panel) for the H + D_2_ → HD(*v*′ = 3, *j*′ = 0) + D reaction at *E*_col_ = 1.97 eV.

Fig. 7 depicts the partial DCS and the *Q*_r_(*θ*,Δ*J*) for three subsets of partial waves that, according to the *J*–*θ* correlation of Fig. 6, can be associated with backward (*J* ∈ [0,10]), sideways (*J* ∈ [11,21]) and forward (*J* > 22) scattering. There is a remarkable similarity between the partial DCSs and the corresponding *Q*(*θ*,[*J*_1_,*J*_2_]) for each of the three intervals, implying that there is essentially no interference between the partial waves belonging to the different subsets. Only at forward scattering angles there is some appreciable interference between partial waves associated with *J* values pertaining to the [11,21] and *J* > 22 intervals. There is one more aspect that deserves a comment. The maxima and minima that can be observed in the DCS can be easily inferred from the positive and negative values of the QM GDF. In particular, the minima at 70°, 115° and 150° correspond to the negative (green colour) contributions in the QM GDF. These minima (and the precedent or subsequent maxima) cannot be deduced from the classical GDF.

**Fig. 8:**
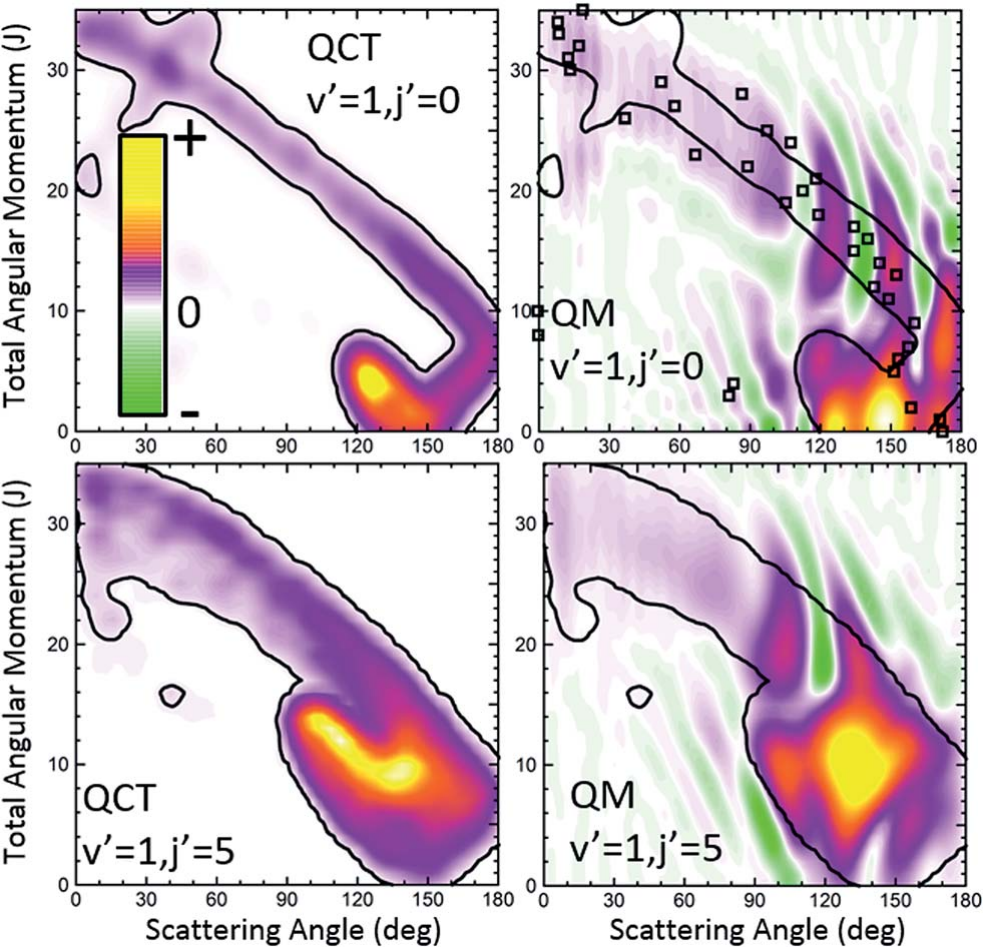
QCT (left) and QM (right) GDFs for the H + D_2_ → HD(*v*′ = 1, *j*′ = 0, 5) + D reaction at *E*_col_ = 1.97 eV. The results for *j*′ = 0 and *j*′ = 5 are shown in the top and bottom panels, respectively. The contours of the classical GDFs are added to the plots representing the QM *Q*_r_(*θ*,*J*) to highlight the similarities and differences. For the HD(*v*′ = 1, *j*′ = 0) formation, the QDF is also represented as open squares.

Let us now move to the collisions leading to HD(*v*′ = 1, *j*′ = 0). The QCT and QM GDFs are shown in the top panels of Fig. 8. As discussed in previous work,^15^ and can be seen by the inspection of the QCT GDF, there are two main, distinct mechanisms that are likely to interact with each other giving rise to the interference pattern observed experimentally. One of them corresponds to the main band with a negative slope, similar to that we have found for *v*′ = 3; the other mechanism, confined in a small region of the *J*–*θ* map, between 110 and 160° and low *J* values, accounts for most of the reactivity. Between them, as a sort of bridge, there is still a third mechanism with a positive slope that comprises low values of *J* and *θ* > 160°. Using the QCT GDF it could be predicted that interference will take place,^15^ since different paths with different *J*s are leading to the same scattering angles. However, the QCT GDF cannot resolve the interference pattern: the number of oscillations and what would be their positions. In previous examples, we have shown that the QM GDFs were akin to their QCT counterparts. In this example, however, we will see that the quantum *Q*_r_(*θ*,*J*) provides additional and most valuable information.

The first observation is that the QM GDF shown in the top-right panel of Fig. 8 is rather different to its classical counterpart. Only with the help of the superimposed contour of the classical *σ*_r_(*θ*,*J*) and leaving aside the destructive coherences, we could see that they share the main gross features. Even then, the QM GDF is broader, and the region corresponding to the diagonal band almost merges with the mechanism confined between 110 and 160° and *J* < 10. But the main source of discrepancy lies in the presence of negative, destructive (green colour) and positive, constructive (red colour) interference that does not flank the main band – as in the case of HD(*v*′ = 3, *j*′ = 0) scattering – but it is transversal to it, cutting the diagonal band into several slices. Since *Q*_r_(*θ*,*J*) is additive, it is easy to realize that each of the slices corresponds to the various peaks in the DCS, whilst the vertical green stripes correspond to minima in the DCS. Therefore, just by looking at the QM GDF we could discern (i) that there will be three peaks in the backward hemisphere, (ii) which will be their positions, as well as those of the respective minima, and (iii) the partial waves that contribute to each of the peaks.

**Fig. 9:**
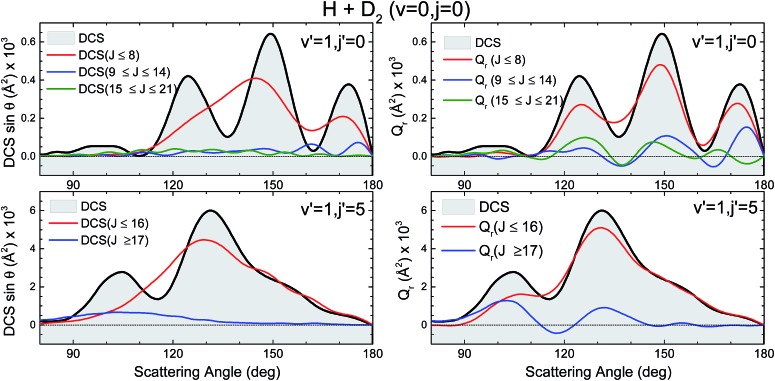
Comparison of the partial DCS (left panels) and *Q*_r_(*θ*,Δ*J*) (right panels) for the H + D_2_ reaction at *E*_col_ = 1.97 eV. Top panels is for scattering giving rise to HD(*v*′ = 1, *j*′ = 0), whilst the bottom panel corresponds to *v*′ = 1, *j*′ = 5. Only the backward hemisphere is shown for clarity.

Not surprisingly, the partial DCS and the QM GDFs summed over a range of *J* values, *Q*_r_(*θ*,Δ*J*), calculated for subsets of partial waves and shown in Fig. 9 do not look alike. The DCS(*J* ≤ 8) can be associated with the confined mechanism and, although it carries most of the reactivity, it shows a broad, blunt shape with no hint of the three finger-like peaks present in the total DCS in the 100–180° range. In stark contrast, the *Q*_r_(*θ*,0 ≤ *J* ≤ 8), that accounts for all the coherences for which the *J* ∈ [0,8] range participates, looks similar to the overall DCS. The partial DCSs calculated for *J* > 8 (*J* ∈ [9,14] and *J* ∈ [15,21]) are very small throughout the whole range of scattering angles, whereas their respective *Q*_r_(*θ*,Δ*J*) is not that small. On top of that, at some angles they are negative, a consequence of the negative contours shown in Fig. 8.

The third scenario corresponds to collisions leading to HD(*v*′ = 1, *j*′ = 5) whose QCT and QM GDFs are portrayed in the bottom panels of Fig. 8. As can be seen, the structure that appeared at low *J*s for HD(*v*′ = 1, *j*′ = 0) has almost merged into the diagonal band and is considerably less confined. In addition, QCT and QM GDFs look now more alike. Yet, the main band is cut by the signature of destructive interference (the green slice at *θ* ∼ 115°) that can be expected to give rise to a minimum in the backward DCS.

The comparison of the partial DCS and the *Q*_r_(*θ*,Δ*J*) confirms these findings and clarifies the role of interference. The choice of *J* = 16 for the decomposition seems to be a sensible choice in light of the deflection functions shown in Fig. 8. In contrast to the results for HD(*v*′ = 1, *j*′ = 0), the DCS(*J* ≤ 16) is similar to *Q*_r_(*θ*,*J* ≤ 16), although the latter is somewhat more structured. However, the *Q*_r_(*θ*,*J* ≥ 17) displays some oscillations and a negative contribution at *θ* ≈ 115° (as expected from the green slice commented on above) which reveals coherences with the low subset of partial waves. The effect of these partial waves is to sharpen the shape of the DCS, defining more clearly the two maxima and the intermediate minimum.

Finally, it is worthwhile to compare the results obtained using the formalism devised in this work with the QDF. In Fig. 6 and 8, superimposed to the *Q*_r_(*θ*,*J*), the respective QDFs for *v*′ = 3, *j*′ = 0 and *v*′ = 1, *j*′ = 0 are represented as open squares. For *v*′ = 3 the agreement is fairly good, covering the regions occupied by the present QM GDF. In particular, the oscillations observed in extreme forward, which could be predicted by the *Q*_r_(*θ*,*J*), can be also foreseen using the QDF (different *J*s leading to the same *θ*). In fact, using the QDF it can be concluded that they are caused by interference between nearside and farside reactive flux.^65^ For the *v*′ = 1 case, however, the sole analysis of the QDF barely accounts for the confined, predominant mechanism. It must be pointed out that even if we could observe the various mechanisms in the QDF, it would not have been possible to predict either the number of peaks and dips or their position since, because of its construction, it only provides one single value of the deflection angle per partial wave.

## 4 Conclusions

The analysis of concurrent reaction mechanisms that govern a chemical reaction can be very challenging, especially in the case of quantum scattering calculations where observables such as the angular momentum and scattering angle are intrinsically entangled. Furthermore, the knowledge of the DCS and the reaction probability as a function of the angular momentum is usually insufficient. One step towards a more thorough understanding of how reactive (or inelastic) collisions take place is to calculate the classical *J*–*θ* joint probability distribution (or the classical generalized deflection function, GDF) since different reaction mechanisms often appear as discontinuities and/or different trends that can be easily visualized in a *J*–*θ* representation. Indeed, from its inspection one can disentangle reaction mechanisms and predict the presence of interference. However, we cannot always rely on classical mechanics, which limits the use of the classical GDF. Therefore, a quantum equivalent of the classical GDF is desirable. While in a classical scheme there is no obstacle in calculating the *J*–*θ* joint probability distribution, devising the same correlation in the QM framework appears unsurmountable due to coherences between different values of *J*; that is, in QM calculations the contribution of several angular momenta to a given *θ* cannot be easily disentangled.

Throughout this article, we propose a conceptually simple quantum GDF, analogous to the classical *J*–*θ* correlation function, which does account for the coherences between *J* partial waves and whose interpretation is rather intuitive. Moreover, the QM GDF presented here not only relates scattering angles to angular momenta but also accounts for the scattering intensity. As such, summing over the whole set of angular momenta for convergence yields the DCS, and integrating over scattering gives the reactive (or inelastic) partial cross section. The calculation of the QM GDF does not require any additional computational effort for the calculation of the DCS or *J* reaction probability.

In this article we have exemplified the proposed QM GDF with several case studies comprising inelastic collisions of Cl + H_2_, the barrierless (and presumably statistical) D^+^ + H_2_ reaction, and the direct H + D_2_ reaction for different final states. Our results show that classical and QM GDFs are essentially coincident whenever quantum interference is not preeminent, although the latter is capable of adding valuable details. When quantum phenomena are present, the QM GDF arises as a powerful tool and makes it possible to observe the interference pattern at first sight and to disentangle the *J* partial waves that contribute to constructive and destructive interference. It also provides information on the number and position of the peaks in the DCS, which something that cannot be extracted from the classical GDF.

The methodology devised here is completely general and can be used to obtain GDF functions for any reaction including those involving more than three atoms. Moreover, due to its quantum mechanical nature, it can be used to analyse reaction mechanisms that do not have a classical analog or under conditions where the classical deflection cannot be calculated, such as at energies below the barrier or whenever either resonances or diffraction phenomena are observed. In summary, we deem the QM GDF as a valuable tool for the analysis of the results obtained using quantum dynamics that can be easily implemented by any researcher in reaction dynamics.

## Conflicts of interest

There are no conflicts to declare.
